# Ultrastructural changes in chronic inflammatory enteropathies—a comparison between dogs and humans

**DOI:** 10.3389/fcell.2024.1379714

**Published:** 2024-05-30

**Authors:** Simone A. Fietz, Mirjam Kalusa, Albert E. Jergens, Dipak Kumar Sahoo, Tracey Stewart, Romy M. Heilmann

**Affiliations:** ^1^ Institute of Anatomy, Histology and Embryology, College of Veterinary Medicine, Leipzig University, Leipzig, Saxony, Germany; ^2^ Department of Veterinary Clinical Sciences, College of Veterinary Medicine, Iowa State University, Ames, IA, United States; ^3^ Department for Small Animals, College of Veterinary Medicine, Leipzig University, Leipzig, Saxony, Germany

**Keywords:** chronic inflammatory enteropathies, inflammatory bowel diseases, ultrastructural changes, dog, human

## Abstract

Chronic inflammatory enteropathies (CIEs) are an important group of diseases in dogs and involve complex pathogenetic aspects. Endoscopy and histopathology are vital for documenting the disease but are less useful for subclassifying CIEs and predicting the response to treatment. However, healing of the mucosal disease process (deep remission) and ultrastructural evaluation of the mucosa have received little attention in canine CIE. Given that canine CIE shares many similarities with inflammatory bowel diseases (IBDs) in human patients—and presents a good spontaneous disease model for human IBD—this perspective article evaluates the literature on ultrastructural lesions in canine CIE and human IBD and offers future directions for the study of ultrastructural mucosal lesions in canine CIE. Such lesions might have a higher sensitivity of detection than structural changes revealed upon light microscopy and may even precede or remain after the resolution of the clinical signs and histologic lesions.

## Chronic intestinal inflammation in dogs and humans

Chronic inflammatory enteropathies (CIEs) are characterized by the clinical characteristics of chronic persistent or recurrent gastrointestinal signs, including vomiting, diarrhea, weight loss, and abdominal pain (Jergens and Heilmann, 2022). The pathogenesis of the disease is complex and involves genetics and environmental factors, resulting in an exacerbated and perpetuated intestinal mucosal immune response (Jergens and Heilmann, 2022). Canine CIE is currently subclassified based on the retrospective assessment of the treatment response and disease remission into either food-responsive enteropathy (FRE), steroid- or immunosuppressant-responsive enteropathy (SRE/IRE), and non-responsive enteropathy (Jergens and Heilmann, 2022). These entities of CIE are histopathologically similar, and the assessment of disease severity is currently primarily based on grading schemes evaluating the clinical and clinicopathological data (Allenspach et al., 2007; Jergens et al., 2010). A special subcategory of CIE is protein-losing enteropathy (PLE) resulting from marked inflammatory infiltration.

Endoscopy and histopathologic assessments are vital for documenting the disease (Washabau et al., 2010; Slovak et al., 2015). These diagnostics are not routinely employed as a monitoring tool, especially as the histologic resolution of duodenal lesions in CIEs was absent in most dogs responding during the induction phase of treatment (Garcia-Sancho et al., 2007; Schreiner et al., 2008). However, the role of mucosal healing (deep remission) has not been extensively investigated in canine CIEs (Jergens and Heilmann, 2022).

Canine CIE shares many similarities with chronic inflammatory enteropathies or inflammatory bowel diseases (IBDs) in human patients (Jergens and Simpson, 2012), comprising Crohn’s disease (CD), ulcerative colitis (UC), and indeterminate inflammatory bowel disease (Beniwal and Harrell, 2010; Ashton and Beattie, 2023). Despite these overlapping disease characteristics, several diagnostic and management features appear to be unique to either canine CIE or human IBD, including the disease location and distribution, immunological signatures such as the cytokine profiles of T helper cells (Heilmann and Allenspach, 2017), and long-term risks (Jergens and Simpson, 2012). However, canine CIE—despite presenting a good spontaneous disease model for human IBD—is generally less studied than human IBD.

## Intestinal ultrastructure

The intestinal tract has the basic structure of a membranous–muscular tube. The luminal mucosa comprises an epithelium overlaying a basement membrane and a loose connective tissue layer (lamina propria mucosae). The underlying lamina muscularis mucosae consist of smooth muscle cells that facilitate the motility of the intestinal mucosa. In the small intestine, mucosal enlargement results from mucosal folds with characteristic finger-like villi of approximately 0.5–1.6 mm in length that protrude into the intestinal lumen and tubular crypts that extend into the intestinal wall (Liebich, 2010; Kummer and Welsch, 2018).

Villi are generally absent on the mucosa throughout the large intestine, but the large intestinal mucosa contains small folds and deep tubular crypts at a high density. The villi and crypts are covered by a single layer of columnar epithelium, mainly consisting of absorptive enterocytes, goblet cells, and few endocrine (Paneth) cells. Enterocytes are characterized by an apical brush border consisting of microvilli. These microvilli have an approximate width of 80 nm, height of 1.0–1.4 µm, and are covered by a 0.3–0.5-µm glycocalyx layer (Marin et al., 1983). However, the abundance of microvilli in the epithelial cells of the large intestine is significantly lower than that in the small intestine (Washabau, 2013). Enterocytes have an oval-shaped nucleus that is located in the basal ⅔ compartment of the cell and is overlaid by the Golgi apparatus, lysosomes, mitochondria, and rough and smooth endoplasmic reticulum (ER). Goblet cells ensure a continuous merocrine release of a glycoprotein- and glycolipid-rich cytoprotective mucin layer. These cells have a narrow base with a basal nucleus and organelles composed of a well-developed ER and Golgi apparatus. The apical part of the goblet cells is typically dilated and filled with mucin droplets, which are membrane-bound and 1–2 µm in diameter.

Epithelial cells are attached to the basal membrane via hemidesmosomes and are firmly linked together by an apical junctional complex composed of—from the apical to basal region—tight junctions (TJs) (zonula occludens), adherens junctions (AJs) (zonula adherens), and desmosomes (macula adherens), providing adhesive and other mechanical properties that seal and restrict the exchange of substances across the paracellular space (Toner, 1968; Demling et al., 1969; Roda et al., 2010; Henderson et al., 2011; Buckley and Turner, 2018).

The submucosa separates the lamina muscularis mucosae from the muscularis propria and contains loose connective tissue with blood vessels, lymphatics, adipose tissue, and scattered immune cells, as well as the submucosal plexus (Meissner plexus), a nerve plexus that controls the secretion and motility of the inner intestinal wall layers. The muscularis propria consists of the inner circular and outer longitudinal layers of smooth muscle cells and a few interstitial cells of Cajal that have an intestinal pacemaker function. Located between these two muscle layers is the myenteric plexus (Auerbach plexus) that regulates the peristalsis of the muscularis layer. The muscularis propria in most areas of the intestinal tract is covered by a single layer of flat epithelium, the serosa, which covers an underlying thin layer of connective tissue (Hoyle and Burnstock, 1989; Ibba-Manneschi et al., 1995; Liebich, 2010; Kummer and Welsch, 2018).

## Ultrastructural disease characteristics of canine CIEs

Ultrastructural lesions can be expected to have a higher sensitivity of detection than structural changes revealed upon light microscopy and may even precede or remain after the resolution of the latter (Sbarbati et al., 2003). However, data on ultrastructural lesions in canine CIE are limited. Abnormalities of the brush border and mitochondrial lesions (cristeolysis and swelling) in endoscopic biopsies of the duodenum have been described in the food-responsive phenotype of canine CIE (Table 1) (Walker et al., 2013). Furthermore, these lesions improved upon clinical remission, showing a reduction in the enterocyte intermicrovillar space and increased microvillus height after 6 weeks of dietary intervention using a hydrolyzed protein diet (Walker et al., 2013). However, potential lesions in the ileum and/or colon have not been evaluated in treatment-naïve dogs with CIE or in those dogs undergoing sequential treatment options (Jergens and Heilmann, 2022), warranting further research into morphometric changes at the subcellular level.

**TABLE 1:** Ultrastructural criteria evaluated in canine chronic inflammatory enteropathy (CIE) (Walker et al., 2013).

Qualitative observations^a^	Quantitative observations^a^
• Mitochondrial lesions	• Microvillus height and diameter
• Microvillar vesiculation	• Intermicrovillar space
• Cytoplasmic vacuolation	• Tight junction width

^a^ Scored as 0, normal; 1, mild; 2, moderate; and 3, severe lesions. Mitochondrial lesions, intermicrovillar space and cytoplasmic vacuolation were significantly altered in CIE at presentation vs. healthy dogs; mitochondrial lesions, microvillus height and intermicrovillar space improved significantly with dietary intervention.

As a breed-specific disease entity separate from canine CIE, gluten-sensitive enteropathy in Irish setters was also revealed to produce microvillus lesions, including a reduction in the size and number, irregularities, and vesiculation.

## Ultrastructural lesions in human IBDs

Differential mucosal abnormalities in IBD (Table 2) included a preserved mucosal integrity with the loss of regular polygonal units and increased mucous production in patients with CD but sloughing and mucosal disintegration with decreased mucous production in UC patients (Trabucchi et al., 1986). Interestingly, these UC lesions could even be observed in endoscopically normal areas of the colon (Trabucchi et al., 1986; Bertini et al., 1998), making electron microscopy (EM) a sensitive tool to detect mucosal lesions and potentially deep remission. In other studies of human IBD, which focused on either CD or UC, the ultrastructural lesions evaluated included several alterations affecting the different structural units of the intestinal wall (Table 2) (Aluwihare, 1971; Ranlov et al., 1972; Dvorak et al., 1979; Myllarniemi and Nickels, 1980; Rickert and Carter, 1980; Lewis et al., 1984; Nyhlin and Stenling, 1984; Dvorak and Silen, 1985; Shields et al., 1985; Nagel et al., 1995; Bertini et al., 1998; Fratila and Craciun, 2010; Zhao et al., 2021; Zhou et al., 2023). While most lesions overlapped between childhood and adult cases of IBDs, some lesions presumed to reflect the chronicity of the disease process (e.g., villus bridging and goblet cell reduction) were primarily observed in adult IBD (Bertini et al., 1998) or were suspected to be specific for childhood IBD (Lewis et al., 1984). As an important differential diagnosis of IBD in humans, irritable bowel disease can also produce ultrastructural lesions (Miglietta et al., 2021). In addition, some overlapping (e.g., microvillus reduction or loss and intermicrovillus space enlargement) and also unique features (e.g., increased mucous layer and pseudomembrane coating of the epithelium) exist compared to chronic infectious diarrhea of bacterial (Fagundes-Neto et al., 2000) or protozoal origin (Poley and Rosenfield, 1982), non-infectious etiologies such as the irritable bowel syndrome (IBS)-like disorders including celiac disease (Shiner and Birbeck, 1961; Miglietta et al., 2021), or even systemic conditions with the potential to affect the integrity of the gastrointestinal barrier, such as an experimental sepsis model (Obermuller et al., 2020).

**TABLE 2:** Summary of the ultrastructural lesions identified in human CD and UC.

Tissue	Lesions in CD	Lesions in UC
Luminal epithelium	• Focal loss of the epithelium (Dvorak and Dickersin, 1979; Dvorak et al., 1979; Marin et al., 1983)	• Focal loss of the epithelium (Delpre et al., 1989)
	• Increased mucus secretion (Dvorak and Dickersin, 1979; Dvorak et al., 1979; Bertini et al., 1998)	• Reduction in the number of crypts and crypt openings (Shields et al., 1985; Bertini et al., 1998)
	• Glycocalyx reduction or loss (Bertini et al., 1998)	• Crypt lesions (deformation, furrows, atrophy, and/or distorted and dilated openings) (Shields et al., 1985; Bertini et al., 1998; Fratila and Craciun, 2010)
	• Crypt opening dilatation (Marin et al., 1983)	• Increase in the number of undifferentiated, immature cells (Mughal and Filipe, 1992)
	• Microulcerations (1–6 cells in diameter) (Marin et al., 1983)	• Enterocytes
	• Increase in the number of undifferentiated, immature cells (Mughal and Filipe, 1992)	- Distortion and loss of hexagonal shape (Kavin et al., 1970; Shields et al., 1985)
	• Enterocytes	- Microvillus alterations (shortening, enlargement, and vacuolization) or loss (Myllarniemi and Nickels, 1980; Delpre et al., 1989; Mughal and Filipe, 1992; Bertini et al., 1998; Fratila and Craciun, 2010)
	- Cell bridging or building (Marin et al., 1983; Nyhlin and Stenling, 1984; Nagel et al., 1995)	- Hyperplasia and/or hypertrophy (Shields et al., 1985; Mughal and Filipe, 1992; Bertini et al., 1998)
	- Loss of hexagonal shape (Myllarniemi and Nickels, 1980; Marin et al., 1983; Nyhlin and Stenling, 1984)	- Large apical lysosomes (Bertini et al., 1998), cytoplasmic vacuolization (Delpre et al., 1989; Fratila and Craciun, 2010), and/or increase in the number of electron-dense vesicles (Mughal and Filipe, 1992)
	- Tight junction fragmentation or loss (Marin et al., 1983)	- ER swelling (Delpre et al., 1989)
	- Microvillus alterations (shortening, thickening, enlargement, and fusion) or loss (Dvorak and Dickersin, 1979, 1980; Dvorak et al., 1979; Rickert and Carter, 1980; Marin et al., 1983; Nyhlin and Stenling, 1984; Nagel et al., 1995; Bertini et al., 1998)	- Golgi zone swelling (Delpre et al., 1989)
	- Abnormal small electron-dense, microvillus- and desmosome-associated bodies (Lewis et al., 1984)	- Irregular, pycnotic nuclei (Mughal and Filipe, 1992; Fratila and Craciun, 2010) with a loosely arranged nuclear membrane (Mughal and Filipe, 1992)
	- Increase in the number of electron-dense lysosomal granules (Ranlov et al., 1972; Dvorak and Dickersin, 1980; Thyberg et al., 1981; Marin et al., 1983)	- Mitochondrial alterations (swelling with disarranged cristae) (Delpre et al., 1989; Fratila and Craciun, 2010)
	- Mitochondrial lesions or pleomorphy (swelling with disarranged cristae) (Ranlov et al., 1972; Mughal and Filipe, 1992; Nazli et al., 2004)	- Pseudopod-like extensions of the cell membrane (Delpre et al., 1989)
	- ER swelling (Ranlov et al., 1972)	- Dilation of the intercellular space (Delpre et al., 1989)
	- Nuclei with a loosely arranged nuclear membrane (Mughal and Filipe, 1992)	• Goblet cells
	• Goblet cells	- Hypoplasia (Kavin et al., 1970; Myllarniemi and Nickels, 1980; Bertini et al., 1998; Fratila and Craciun, 2010) or hyperplasia (Mughal and Filipe, 1992)
	- Orifice dilation (Dvorak and Dickersin, 1979; Dvorak et al., 1979; Marin et al., 1983)	- ER swelling (Fratila and Craciun, 2010)
	- Hyperplasia and/or hypertrophy (Dvorak et al., 1979; Myllarniemi and Nickels, 1980; Marin et al., 1983; Nagel et al., 1995)	- Heterogeneity (Mughal and Filipe, 1992) and decrease (Delpre et al., 1989) of mucin droplets
	- Immature mucigenic granules (Dvorak and Dickersin, 1980)	
	- Abnormal small electron-dense, microvillus- and desmosome-associated bodies (Lewis et al., 1984)	
	• Paneth cells	
	- Irregular lysosomal inclusions/increased granule formation in Golgi areas (Dvorak and Dickersin, 1980)	
	- Hyperplasia (Dvorak and Dickersin, 1980)	
Connective tissue and vasculature	• Extracellular edema with marked infiltrates of inflammatory cells (Ranlov et al., 1972; Thyberg et al., 1981; Brewer et al., 1990)	• Marked infiltrates of inflammatory cells (Delpre et al., 1989; Brewer et al., 1990)
	• Activated fibroblasts and extracellular fibrin deposition (Ranlov et al., 1972)	• Lymphatics
	• Fragmented or irregularly arranged collagen (Dvorak et al., 1980b)	- Intracellular edema in endothelial cells (Aluwihare, 1971)
	• Lymphatics	
	- Distension (Dvorak et al., 1980a)	
	- Large inter-endothelial gaps (Dvorak et al., 1980a)	
	• Arterioles and arteries	
	- Intimal proliferation (Dvorak et al., 1980a)	
	- Fragmented elastic tissue (Dvorak et al., 1980a)	
	- Increased adventitial collagen (Dvorak et al., 1980a)	
	• Capillaries	
	- Endothelial swelling (Brewer et al., 1990)	
	• Venules	
	- Lumen filled with platelets and fibrin strands (Dvorak et al., 1980a)	
	- Focal endothelial necrosis (Dvorak et al., 1980a)	
	- Endothelial cells and pericytes with non-membrane-bound lipid bodies (Dvorak et al., 1980a)	
	- Basal lamina reduplications	
	• Macrophages	
	- Increase in the number of electron-dense lysosomal granules (Thyberg et al., 1981)	
	• Mast cells	
	- Granule alterations (size, number, and density/degranulation) (Dvorak et al., 1980a; Wang et al., 2007)	
	- Elongated surface villi (Dvorak et al., 1980a)	
	• Basophils	
	- Degranulation (Dvorak et al., 1980a)	
	• Eosinophils	
	- Granule alterations (size, number, and density) (Dvorak et al., 1980a)	
	• Lymphocytes	
	- Prominent nucleoli (Aluwihare, 1971)	
	• Lymphoid cells	
	- Poly-ribosomal structures (Ranlov et al., 1972)	
	- Golgi zone enlargement (Ranlov et al., 1972)	
Musculature	• Smooth muscle cells	• Interstitial cells of Cajal
	- Hyperplasia and/or hypertrophy (Dvorak et al., 1980b)	- Lipid droplets (Rumessen, 1996; Rumessen et al., 2010)
	- Necrosis (Dvorak et al., 1980b)	- Disrupted glycogen vacuoles (Rumessen, 1996) and irregular vacuoles (Rumessen et al., 2010)
	- Cytoplasmic vacuoles with collagen fibers or lipids (Dvorak et al., 1980b)	
	- Hypercontraction (Dvorak et al., 1980b)	
	- Myofibroblastic transformation (Dvorak et al., 1980b)	
	• Stellate cells	
	- Dilated Golgi area (Dvorak et al., 1980b)	
	- Increase in the rough ER (Dvorak et al., 1980b)	
	• Interstitial cells of Cajal	
	- Secondary lysosomes and vacuolization (Wang et al., 2007; Rumessen et al., 2011)	
	- Disrupted vacuoles associated with the rough ER and glycogen clumps (Rumessen et al., 2011)	
	- Mitochondrial swelling or loss (Wang et al., 2007)	
	- Lipid droplets (Wang et al., 2007)	
	- Cytoplasmic filament reduction or loss (Wang et al., 2007)	
	- Perinuclear damage (Wang et al., 2007)	
Autonomic nervous system	• Nerve trunk enlargement and distortion (Dvorak et al., 1980b)	• Axons
	• Axons	- Damage (myelin figures, microtubule reduction, and terminal swelling) or necrosis (swollen and empty axons) (Brewer et al., 1990; Dvorak et al., 1993; Rumessen, 1996; Geboes and Collins, 1998; Rumessen et al., 2010)
	- Damage (myelin figures, microtubule reduction, terminal swelling, and lipid droplets) or necrosis (swollen, empty axons) (Dvorak et al., 1980b; Dvorak and Silen, 1985; Steinhoff et al., 1988; Brewer et al., 1990; Dvorak et al., 1993; Geboes and Collins, 1998; Wang et al., 2007; Rumessen et al., 2011)	
	- Mitochondrial swelling (Dvorak et al., 1980b)	
	- Large membrane-bound vacuoles (Dvorak et al., 1980b)	
	- Dense core granules (Dvorak et al., 1980a)	
	- Neurofibril accumulation (Dvorak et al., 1980b)	
	• Synaptic membrane thickening (Dvorak et al., 1980b)	
	• Ganglion cell enlargement with an increase in the rough ER (Dvorak et al., 1980b)	

The intestinal epithelial cell ultrastructure was also altered in experimental rodent models of IBD (Pfeiffer et al., 1997; Tian et al., 2003; Bou-Fersen et al., 2008; Bochimoto et al., 2019). The colonic ultrastructure in a 2,4,6-trinitrobenzene sulfonic acid (TNBSA)-induced IBD/colitis model, for example, revealed a mixed picture of deformed intestinal crypt areas with high cell migration rates and a more regular structure with low cell migration rates in the remaining intestinal crypts (Bochimoto et al., 2019). The depletion of goblet cell mucin, stacked to curled Golgi apparatus in absorptive cells, and remnants of the ER support that ER stress plays an important role in the pathogenesis of IBD (Bochimoto et al., 2019).

Some characteristics, such as axonal degeneration or necrosis (Dvorak and Silen, 1985), might be an important feature and pathogenic correlation in IBD but require tissue biopsies that extend at least to the level of the intestinal submucosa. However, this depth is usually not reached with routine endoscopic biopsies (Day et al., 2008; Willard et al., 2010).

## Perspective

Lesion evaluation in human IBD and experimental animal models has traditionally focused on the enterocyte and typically includes the evaluation of the lesions that reflect changes in mitochondrial size and integrity, cytoplasmic injury, microvillus mass, and disruption of tight junctions (Mughal and Filipe, 1992). However, other structures of the mucosa might be equally important to be examined. Transmission EM (TEM) or scanning EM (SEM) could present a useful adjunct tool to evaluate the structures and detect lesions or patterns of tissue regeneration that remain undetected during routine histology (Laschi et al., 1987; Schattenfroh et al., 1994; Bertini et al., 1998). EM is an important tool in medical research and diagnostics, and this technique is usually accessible in most universities and research laboratories.

**FIGURE 1:**
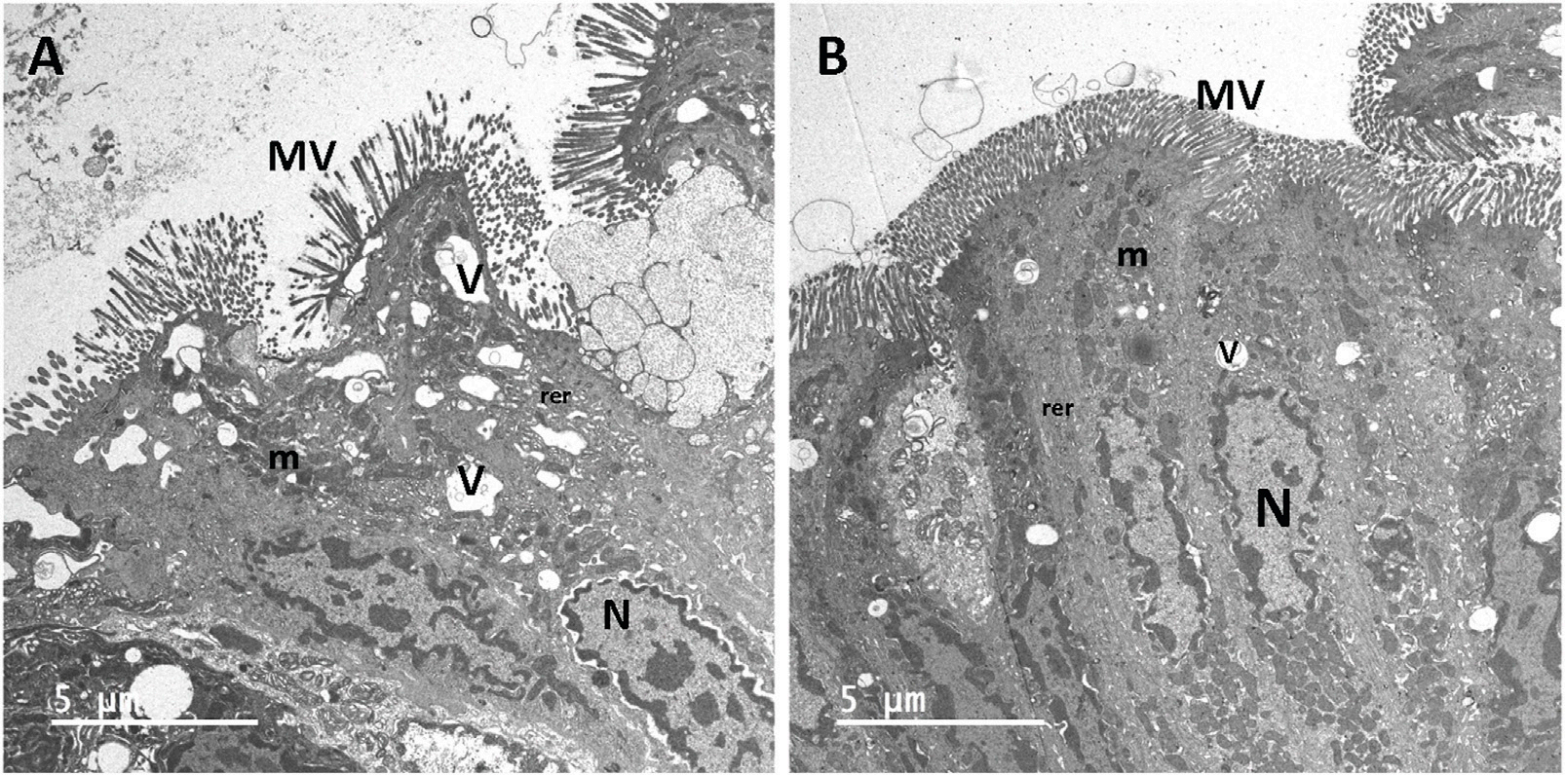
Transmission electron micrographs of enterocytes lining the colon from a dog with chronic inflammatory enteropathy before **(A)** and after **(B)** treatment with synbiotics. Before synbiotic treatment **(A)**, microvilli (MV) are dispersed and vary in size. Large vacuoles (V) show disruptions in the cytoplasm and distension of the rough endoplasmic reticulum (rer). Following synbiotic treatment **(B)**, MV are densely uniform in size, and the enterocytes are markedly less disrupted. The nuclei (N), mitochondria (m), rer, and small vacuoles (v) appear normal.

Very little information about ultrastructural lesions is available for canine CIEs (Walker et al., 2013). Morphological characteristics yet to be investigated in canine CIEs using TEM and/or SEM include any features and particularly goblet cells (and their subcellular mucin droplets) in the colon, any structural lesions in the ileum, mucosal structures other than the enterocyte, and a number of other enterocytic subcellular structures (e.g., ER, Golgi complexes, desmosomes, nuclei, and nucleoli) and cellular lesions including 1) junctional complexes (JCs) focused on TJs, AJs, and desmosomes; 2) autophagic bodies; 3) evidence of apoptosis or necrosis; 4) lipid/chylomicron droplets; 5) other particles (including viral or phage structures); and 6) other lesions shown in human IBD (Table 2). In addition, ultrastructural changes in the response to treatment (or lack thereof) other than dietary intervention—including immunomodulatory treatment or alternative therapeutic options such as pre-/pro-/synbiotics, fecal microbiota transplantation, cholestyramine as bile acid sequestrant, or stem cell therapy (Jergens and Heilmann, 2022)—remain to be studied. In this regard, synbiotic treatment decreases the dispersion and size variation of microvilli and the disruption of enterocytes in a dog with CIE (Figure 1) (Sahoo et al., 2022). Given the number and complexity of possible ultrastructural alterations (Table 2), their detection, grading, and association with other patient and disease characteristics might benefit from using a machine learning algorithm. Deep machine learning might also allow researchers to identify and compare structural lesions along the gastrointestinal tract, follow and integrate longitudinal changes over a more extended time, and utilize an unsupervised convoluted neural network approach to identify currently underestimated or even undetected lesions (Syed and Stidham, 2020; Javaid et al., 2022; Zand et al., 2022). Despite the tedious preparative steps involved in electron microscopy (Laschi et al., 1987), this method shows potential to investigate and integrate still unknown or undetected aspects of canine CIE, especially in the context of its pathogenesis, diagnosis, and response to treatment. A better characterization and deeper understanding of the ultrastructural mucosal changes in canine CIE will ultimately lead to a better definition of the similarities and differences between human IBD and canine CIE and, thus, will shed more light on the suitability of the dog as a spontaneous animal model for human IBD.

## Data Availability

The raw data supporting the conclusion of this article will be made available by the authors, without undue reservation.
